# Feeding neurons integrate metabolic and reproductive states in mice

**DOI:** 10.1016/j.isci.2023.107918

**Published:** 2023-09-14

**Authors:** Megan G. Massa, Rachel L. Scott, Alexandra L. Cara, Laura R. Cortes, Paul B. Vander, Norma P. Sandoval, Jae W. Park, Sahara L. Ali, Leandro M. Velez, Huei-Bin Wang, Shomik S. Ati, Bethlehem Tesfaye, Karen Reue, J. Edward van Veen, Marcus M. Seldin, Stephanie M. Correa

**Affiliations:** 1 Department of Integrative Biology and Physiology, University of California – Los Angeles, Los Angeles, CA 90095, USA; 2 Neuroscience Interdepartmental Doctoral Program, University of California – Los Angeles, Los Angeles, CA 90095, USA; 3 Department of Human Genetics, David Geffen School of Medicine at UCLA, Los Angeles, CA 90095, USA; 4 Department of Biological Chemistry, School of Medicine, University of California – Irvine, Irvine, CA 92697, USA

**Keywords:** Physiology, Neuroscience, Cellular neuroscience

## Abstract

Balance between metabolic and reproductive processes is important for survival, particularly in mammals that gestate their young. How the nervous system coordinates this balance is an active area of study. Herein, we demonstrate that somatostatin (SST) neurons of the tuberal hypothalamus alter feeding in a manner sensitive to metabolic and reproductive states in mice. Whereas chemogenetic activation of SST neurons increased food intake across sexes, ablation decreased food intake only in female mice during proestrus. This ablation effect was only apparent in animals with low body mass. Fat transplantation and bioinformatics analysis of SST neuronal transcriptomes revealed white adipose as a key modulator of these effects. These studies indicate that SST hypothalamic neurons integrate metabolic and reproductive cues by responding to varying levels of circulating estrogens to modulate feeding differentially based on energy stores. Thus, gonadal steroid modulation of neuronal circuits can be context dependent and gated by metabolic status.

## Highlights

• Activation of somatostatin neurons increased feeding across sex
• Somatostatin neuron ablation decreased feeding in low body mass, proestrus females
• Tuberal nucleus somatostatin neurons are sensitive to estrogens and adipokines
• Somatostatin neurons integrate reproductive and metabolic cues to alter feeding

## Graphical abstract

**Figure:**
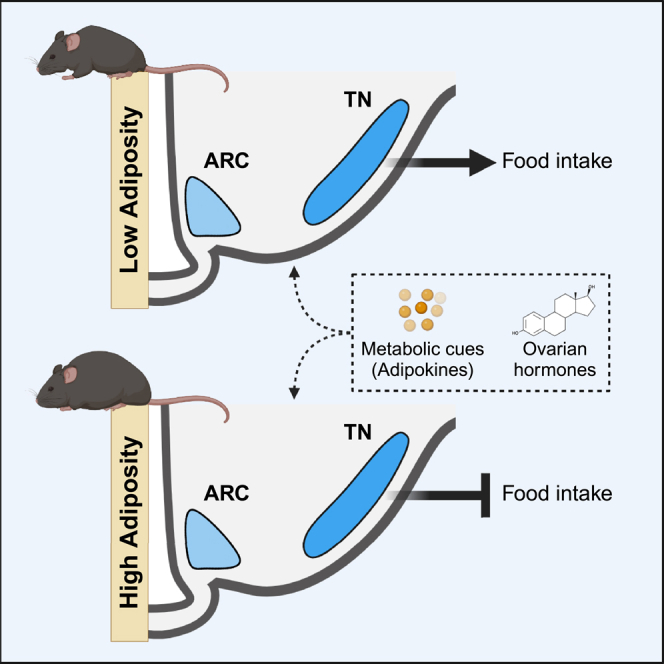

## Introduction

The homeostatic processes of metabolism and reproduction are mutually dependent on one another. Reproductive milestones, including pregnancy and quiescence, result in major metabolic shifts,^1^^,^^2^^,^^3^ and metabolic status can gate reproductive function in menstrual and estrual mammals. Pubertal onset has been shown to require a critical threshold of body fat,^4^^,^^5^ and increasing adiposity is associated with a decreasing age of pubertal onset in individuals with ovaries in particular.^6^ During reproductive years, undernutrition can acutely disrupt menstrual cyclicity^7^ via hypogonadotropic hypogonadism^8^ and result in fewer successful pregnancies.^9^ Rodent models have been used to investigate the effects of sex variables on metabolic tissues. Estradiol has been shown to regulate key metabolic processes such as adiposity,^1^ thermogenic capacity and locomotion,^1^^,^^10^^,^^11^^,^^12^^,^^13^ and feeding,^14^ while sex chromosome complement has been demonstrated to affect fat deposition and energy output.^15^ However, few studies have investigated the mechanisms by which both metabolic and reproductive status reciprocally interact to modulate behavior.

A candidate region for the seat of this interaction is the tuberal hypothalamus, the central region of the hypothalamus typically thought to be comprised of the mediobasal hypothalamus (arcuate nucleus, ventromedial nucleus, and median eminence), dorsomedial nucleus, lateral hypothalamic area, and the lateral tuberal nucleus (TN) (N.B.: to prevent confusion, references to the specific lateral tuberal nucleus are abbreviated TN, whereas “tuberal hypothalamus” and “tuberal hypothalamic” refer to the greater anatomical spatial designation -- the “middle” of the hypothalamus on the rostral-caudal axis -- encompassing the listed nuclei.). Positioned near the third ventricle and partially outside of the blood–brain barrier,^16^ the mediobasal hypothalamus is situated as a nexus region with the ability to sample circulating homeostatic hormones and relay relevant information to other regions of the brain. Importantly, both this region and the greater tuberal hypothalamus are sensitive to and vital for both reproductive and metabolic homeostasis. For instance, in addition to the role of kisspeptin neurons in the arcuate nucleus as key modulators of estrogen-mediated negative feedback in the hypothalamic-pituitary-ovarian axis,^17^^,^^18^^,^^19^ they also require epigenetic de-repression for menarche.^20^^,^^21^ The arcuate nucleus is also home to the canonical homeostatic feeding neurons—appetitive agouti-related peptide (AgRP)/neuropeptide Y (NPY) neurons and satiety-related proopiomelanocortin neurons—which can not only detect and respond to sensory and metabolic cues including leptin, ghrelin, and insulin^22^^,^^23^^,^^24^ but also regulate non-feeding metabolic processes such as bone mass^25^ and insulin resistance via modulation of brown adipose.^26^ The ventromedial nucleus of the hypothalamus, and in particular the ventrolateral subregion, is important for various functions across sexes, including mating behavior (reviewed by Kammel and Correa^27^). Distinct, estrogen-sensitive cellular populations in the ventrolateral ventromedial nucleus contribute to various aspects of metabolism, including locomotion^10^^,^^28^^,^^29^ and thermoregulation.^30^ Given the substantive neuronal heterogeneity within a small brain region, it is unsurprising that these populations form complex functional interactions. For example, simulation of starvation via chronic chemogenetic activation of appetitive arcuate AgRP neurons has been demonstrated to acutely disrupt estrous cyclicity,^31^ providing a neuronal contributor to the link between metabolic state and reproductive function.

Somatostatin (SST) is a key neuropeptide long known to be involved in the regulation of feeding.^32^^,^^33^^,^^34^^,^^35^^,^^36^^,^^37^^,^^38^^,^^39^^,^^40^^,^^41^ Its expression marks many distinct populations within the tuberal hypothalamus. Two such prominent populations are seen in the arcuate nucleus (ARC) and TN. While the ARC is an uncontested member of the mediobasal hypothalamus, the TN straddles the mediobasal hypothalamus and lateral hypothalamic area. It is an understudied region marked by expression of SST, which has been mostly characterized in rats. Somatostatin neurons in both the ARC (ARC^SST^) and TN (TN^SST^) regulate food intake,^42^^,^^43^^,^^44^^,^^45^ and SST expression within the greater hypothalamus is regulated by circulating gonadal steroids.^46^ Studies across various animals have found that ARC^SST^ neurons both colocalize with estrogen receptor^47^ and seem to be responsive to estrogens.^48^ While the TN has not been directly analyzed for gonadal steroid receptor presence, studies have found the TN to be closely apposed to two highly estrogen-sensitive regions, ventrolateral region of the ventromedial hypothalamus^49^ and the lateral hypothalamic area,^42^ suggesting that the TN may also be estrogen sensing.^49^^,^^50^^,^^51^^,^^52^^,^^53^ Furthermore, SST signaling in the ARC influences or can be influenced by the hypothalamic-pituitary-gonadal axis across a variety of species.^47^^,^^48^^,^^54^^,^^55^ Thus, SST neurons in these regions are excellent prospective candidates for integrating metabolic and reproductive cues to affect feeding based on anatomical location, sensitivity to circulating reproductive hormones, detection of metabolic hormones such as ghrelin,^43^ and promotion of feeding behavior. Indeed, whole-body knockouts of SST exhibit weight gain that is exacerbated by sex category and high-fat diet.^56^

Here, we use mice to interrogate the role that SST neurons of tuberal hypothalamus play in integrating metabolic and reproductive cues to affect feeding. SST neurons exhibited differential control of feeding in female and male mice (defined by anogenital distance at weaning and postmortem inspection of the gonads), with neuronal ablation decreasing food intake only in females. This effect was primarily due to a decrease in food intake during proestrus, when circulating ovarian hormones are at high concentrations, and was only observed in animals with a low body mass. To determine whether adiposity could mediate the effect of body mass on food intake, fat transplantation experiments were performed. Increased white adipose tissue was sufficient to alter SST neuronal modulation of food intake. Together, these data reveal a context-dependent role for SST neurons of the tuberal hypothalamic region in the regulation of food intake, by which SST neurons tune feeding behavior in response to metabolic and reproductive states.

## Results

### Chemogenetic activation of TN^SST^ neurons increases food intake in female and male mice

**Figure 1:**
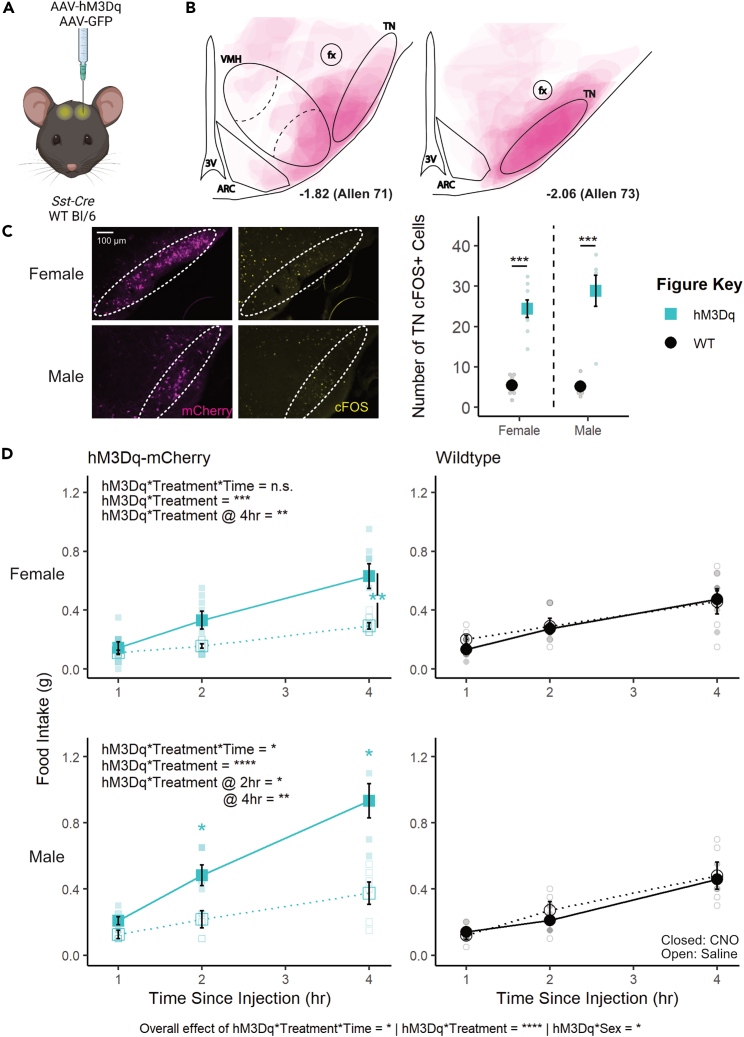
Transient activation of TN^SST^ neurons increased food intake across sexes (A) Schematic of experimental paradigm. AAVs encoding hM3Dq-mCherry or GFP within flip-excision (FLEX) cassettes were injected bilaterally into the lateral tuberal nucleus (TN) of *Sst-Cre* or wild-type mice. Created with BioRender.com. (B) Composite schematic of hM3Dq-mCherry expression. (C) Fluorescent images of mCherry (magenta) and cFOS (yellow) in the TN of *Sst-Cre* mice and quantification of cFOS+ cells in these TN 90 min after CNO injection. Mice infected with h3MDq show an increase of cFOS-positive cells 90 min after CNO injection regardless of animal sex (overall effect of hM3Dq-mCherry presence, F(1,21) = 73.634, p < 0.0001; no interaction effect, F(1,21) = 0.939, p = 0.3435). Dashed ovals indicate boundaries of the TN. Scale bar = 100 μm. (D) Activation of TN^SST^ neurons in both female and male mice leads to higher food intake within the 4-h daytime testing period (left column, interaction between hM3Dq-mCherry presence and treatment: F(2,105) = 3.2964, p = 0.0409). Within sex, only males exhibited an effect of activation and treatment over time (F(2,45) = 3.2793, p = 0.0468), though both females and males exhibited an effect of hM3Dq presence and treatment independent of time (F(1,60) = 12.7928, p = 0.0007 and F(1,45) = 25.1794, p < 0.0001, respectively). Males also exhibited a significant hM3Dq-by-treatment interaction at 2 h post-CNO (F(1,9) = 8.69, p = 0.0163). CNO did not affect food intake in wild-type control mice (right column). Mean ± SEM; ANOVA and post hoc t tests where applicable ∗p < 0.05, ∗∗p < 0.01, ∗∗∗p < 0.001, ∗∗∗∗p < 0.0001. F Control n = 6; M Control n = 5, F hM3Dq n = 8; M hM3Dq n = 6.

To test the role of TN^SST^ neurons across sexes, an adeno-associated virus (AAV) expressing a Cre-dependent Gq-coupled hM3Dq^57^ was stereotaxically injected to the TN of *Sst-Cre* mice (Figures 1A–1C). Overall, activation of TN^SST^ neurons using the small-molecule ligand clozapine-N-oxide (CNO) increased daytime food intake over a 4-h testing period in both females and males, with no sex differences apparent (hM3Dq∗treatment∗time∗sex, F(2,105) = 0.5982, p = 0.5517; Figure 1D). Control animals without expression of hM3Dq-mCherry confirmed no effect of CNO alone, while within-subjects comparisons of animals expressing hM3Dq in TN^SST^ neurons indicated an increase in feeding upon CNO-induced cellular activation. There was a significant interaction between hM3Dq presence and treatment (saline v. CNO), both over time (treatment∗hM3Dq∗time, F(2,105) = 3.2964, p = 0.0409) and irrespective of time (treatment∗hM3Dq, F(1,105) = 35.2054, p < 0.0001). The effect of neuronal activation (genotype-by-treatment interaction) was most prominent across sexes at 4 h post-CNO injection (females: F(1,12) = 10.0208, p = 0.0081; males: F(1,9) = 12.1521, p = 0.0069), though males also exhibited a significant activation-by-treatment interaction at 2 h post-CNO administration (F(1,9) = 8.6957, p = 0.0163). Post hoc within-subjects analyses of animals bilaterally transduced with hM3Dq-mCherry indicated a significant increase in food intake during activation by CNO as compared to treatment with saline control (females overall: t(31) = 2.8486, p = 0.007732; males at 2 h: t(5) = 2.9701, p = 0.0311; males at 4 h: t(5) = 3.3263, p = 0.0286). Thus, activating TN^SST^ neurons elicits feeding across sexes.

### Caspase ablation of SST neurons decreases food intake only in females

**Figure 2:**
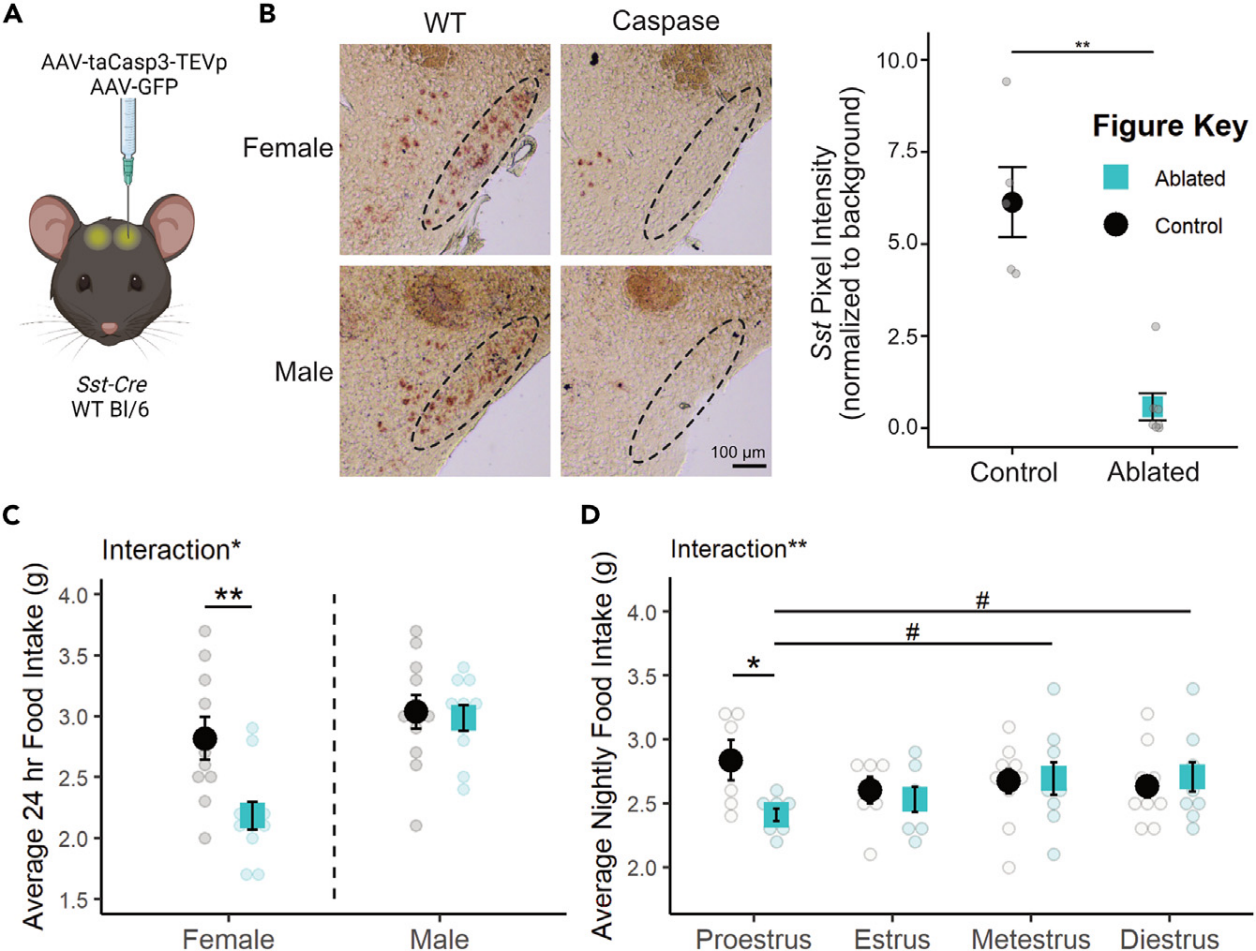
Caspase ablation of SST neurons decreased food intake only in females (A) Schematic of experimental paradigm. AAVs encoding taCasp3-TEVp or GFP within flip-excision (FLEX) cassettes were injected bilaterally into the TN of *Sst-Cre* or wild-type mice. Created with BioRender.com. (B) Representative bright-field images and quantification of *Sst* transcript expression in the TN of caspase-ablated and control animals. Dotted line indicates the boundary of the TN. Scale bar = 100 μm. Viral spread to ARC is quantified in Figure S1A. (C) Permanent SST neuronal ablation decreases average daily food intake in females but not males. F Control n = 10; M Control n = 11; F Ablated n = 11; M Ablated n = 10. (D) This decrease in food intake is detected only in the night of proestrus. Proestrus: Control n = 6, Ablated n = 8; Estrus: Control n = 7, Ablated n = 7; Metestrus: Control n = 10, Ablated n = 9; Diestrus: Control n = 10, Ablated n = 9. Mean ± SEM; ANOVA and post hoc t tests where applicable; between subjects: ∗p < 0.05, ∗∗p < 0.01; within subjects: ^#^p < 0.10.

To determine if permanent inactivation of SST neurons in tuberal hypothalamic regions alters feeding across sexes, an AAV expressing a Cre-dependent modified caspase virus (taCasp3-TEVp^58^) was stereotaxically delivered to the TN of *Sst-Cre* mice (Figure 2A). Bilateral elimination of *Sst* expression was validated by *in situ* hybridization and pixel intensity quantification (t(5.24) = 5.44, p = 0.00247; Figure 2B). While ablation was largely targeted to the TN, there was also a significant decrease in *Sst* stain in the ARC (ablation-by-region interaction: F(3,36) = 7.31388, p = 0.001; post hoc TN t(5.24) = 5.44, p_adj_ = 0.00988; post hoc arcuate t(10) = 3.52, p_adj_ = 0.0167; Figure S1A). Mice were subjected to two 96-h food assays along with a battery of other metabolic tests. Final food intake, accounting for spillage, is depicted as an average over 24 h (Figure 2C). ANOVA revealed an overall effect of sex where males consume more food than females, as expected (F(1,38) = 14.1896, p = 0.0006).

Interestingly, the effect of SST neuron ablation differed by sex (sex-by-ablation interaction: F(1,38) = 4.6852, p = 0.0368), an effect which post hoc t tests revealed to be carried by a decrease in food intake specifically in females (t(15.671) = -3.0561, p = 0.007686, Figure 2C). This sex difference was not previously reported, but collapsing these data across sex results in an overall decrease in food intake with SST neuron ablation (t(39.86) = -2.2713, p = 0.0286), consistent with previous studies.^43^

In order to ensure this effect is due to ingestion and not repetitive motion such as gnawing,^42^ crumb mass was compared across groups. There was an overall effect of sex (F(1,38) = 7.16394, p = 0.0109) such that female mice produced more crumbs on an average day than male mice (Figure S1B). However, there was no effect of genotype (F(1,38) = 3.14328, p = 0.0843) or interaction between sex and genotype (F(1,38) = 2.70519, p = 0.1083) on crumb mass.

The female-specific effect of SST neuron ablation is modulated by estrous cycle stage. There was a significant interaction between SST neuron ablation and estrous stage (F(1,45) = 8.1581, p = 0.0065) which was predominantly due to a decrease in food intake specifically during the night of proestrus (t(5.902) = -2.6044, p = 0.04104, Figure 2D). Within-subjects analysis of mice in the neuronal ablation group suggested that nighttime food intake during proestrus was also slightly lower than consumption during metestrus (t(7) = -1.976, p = 0.0887) and diestrus (t(7) = -2.3276, p = 0.0528), although these effects across the cycle did not reach statistical significance.

Despite effects on food intake, no other metabolic measures were altered by TN^SST^ neuron ablation (see Table S1 for statistical results). SST neuron ablation did not affect telemetry measures of activity/movement (Figure S1C) or core body temperature (Figure S1D), or response to fasting glucose tolerance test (Figure S1E). SST neuron ablation did not affect body mass (Figure S1F), suggesting that the selective decrease in food intake during proestrus was not sufficient to alter body mass.

### Body mass, specifically adiposity, influences the effect of SST neuronal ablation on food intake

**Figure 3:**
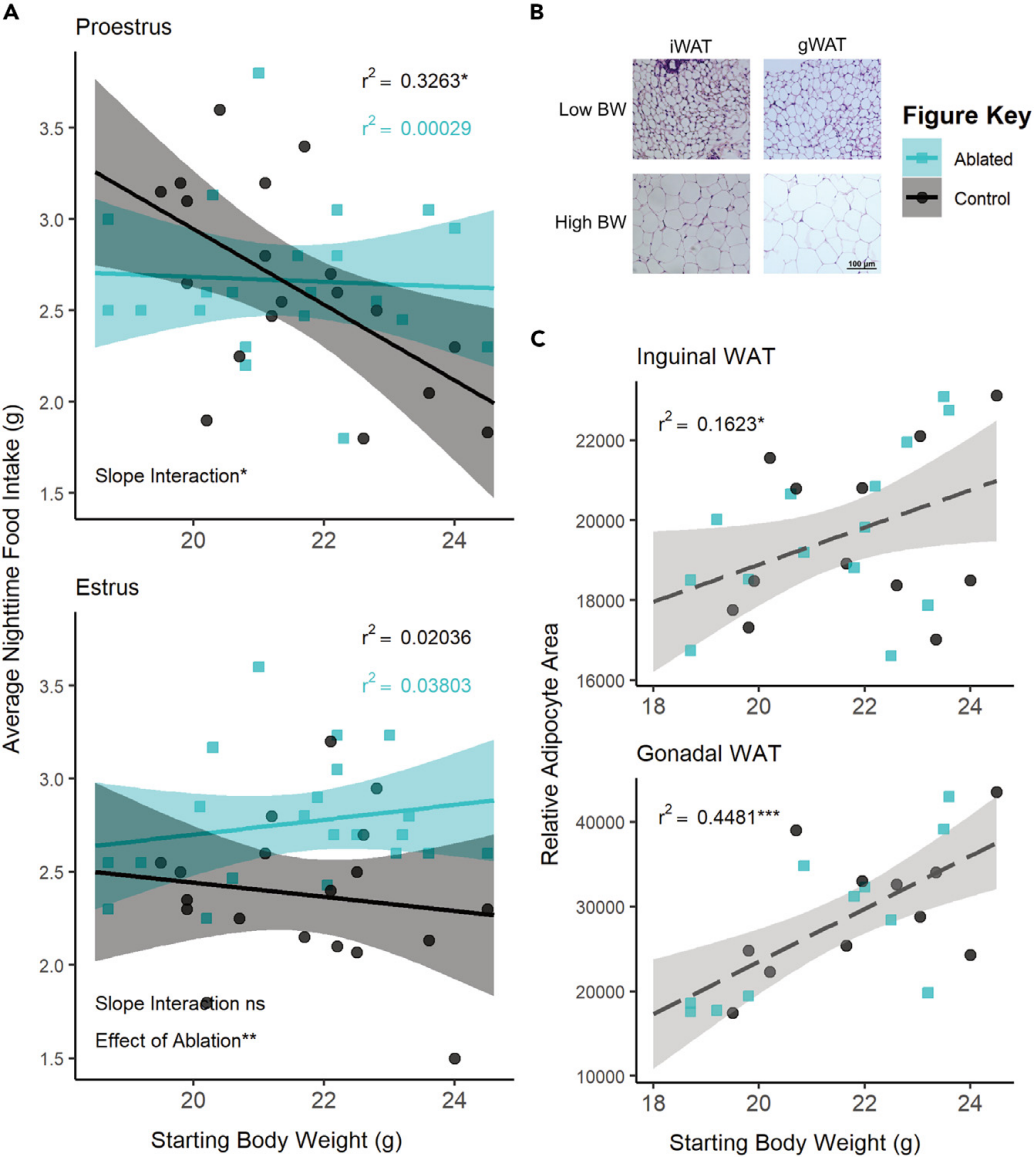
The effect of SST neuron ablation in proestrus depends on body mass or adiposity (A) Regression analysis within proestrus (top panel) and estrus (bottom panel) across all ovary-intact animals reveals an interaction between body mass and nightly food intake in females. Significant negative correlations in wild-type animals are seen in the high-estradiol phase of proestrus but not in caspase-ablated females. Control n = 19, Ablated n = 21 across stages. (B) Representative bright-field images of inguinal and gonadal white adipose tissue (iWAT & gWAT, respectively) in high and low body weight (BW) females. Scale bar = 100 μm. (C) Terminal iWAT (left) and gWAT (right) adipocyte size both positively correlate with starting body weight regardless of TN^SST^ neuron ablation (Interaction of slopes: iWAT F(1,22) = 0.2337 p = 0.6336, gWAT F(1,18) = 0.4552 p = 0.5085; difference in y-intercept: iWAT F(1,22) = 0.1417 p = 0.7103, gWAT F(1,18) = 0.000 p = 9971). Linear regression lines represent compiled data across ablation status. iWAT: Control n = 12, Ablated n = 14; gWAT: Control n = 11, Ablated n = 11. Linear regression ±95% CI; ANCOVA; ∗p < 0.05, ∗∗p < 0.01.

There was an overall interaction between body mass and estrous phase (F(1,150) = 3.9433, p = 0.04889) and between body mass and ablation (F(1,150) = 16.9924, p < 0.0001; see Table S1 for full results). Analyzing the relationship between body mass and food intake within each estrous stage revealed significant negative correlations between body mass and food intake in wild-type animals during nights of proestrus (r^2^ = 0.3263; F(1,17) = 8.235, p = 0.01062; Figure 3A) and metestrus (r^2^ = 0.2801; F(1,16) = 6.227, p = 0.0239; Figure S2A), stages with higher relative circulating estradiol levels.^59^ There were no correlations between food intake and body mass in estrus and diestrus, stages with lower relative circulating estradiol levels (Figure 3A; Figure S2A; Table S1). SST neuron ablation uncoupled this relationship with body mass in high-estradiol stages (proestrus: r^2^ = 0.00029, F(1,19) = 0.05492, p = 0.08172; metestrus: r^2^ = 0.09921, F(1,19) = 2.093, p = 0.1643), suggesting that this neuronal population is required for the body mass-dependent modulation of food intake during these stages.

Ovariectomy to test the influence of ovarian secretions failed to replicate the effects of SST neuron ablation. We did not detect an interaction between ablation and gonad status (F(1,69) = 0.0109, p = 0.9173, Figure S2B), but overall food intake was lower in ovariectomized mice (F(1,69) = 4.4746, p = 0.038, Figure S2B). Body weight was not associated with food intake in either ablated or control animals (24 h: F(1,34) = 0.0076, p = 0.9311; Nightly: F(1,34) = 1.1595, p = 0.289147; Figure S2C), though examining nightly food intake only revealed an effect of ablation status on food intake overall (F(1,34) = 8.4044, p = 0.006512; Figure S2C). Given the influence of body weight on the effect of SST ablation (Figure 3A), it is possible that the lack of an effect in sham mice was at least partially due to higher body weight and altered metabolic profiles in this experiment.

Since fat mass is known to influence feeding, adiposity was examined postmortem. Postmortem adipocyte size of subcutaneous inguinal and visceral perigonadal white adipose tissue (iWAT and gWAT, respectively) positively correlated with the body mass determined at the onset of feeding assays (iWAT: F(1,22) = 4.3346, p = 0.04919; gWAT: F(1,18) = 14.9872, p = 0.001119; Figures 3B and 3C) regardless of SST neuron ablation (ANCOVA revealed no significant interaction of slopes or difference in y-intercept, see Table S1). Body mass accounted for a larger percentage of variation in visceral gWAT adipocyte size (r^2^ = 0.4481; F(1,20) = 16.24, p = 0.0006557) than it did for subcutaneous iWAT (r^2^ = 0.1623; F(1,24) = 4.649, p = 0.0413), suggesting a possible increased contribution of visceral adiposity to the effect of TN^SST^ neuron ablation.

### TN^SST^ neurons are sensitive to estrogens and adiposity signals

**Figure 4:**
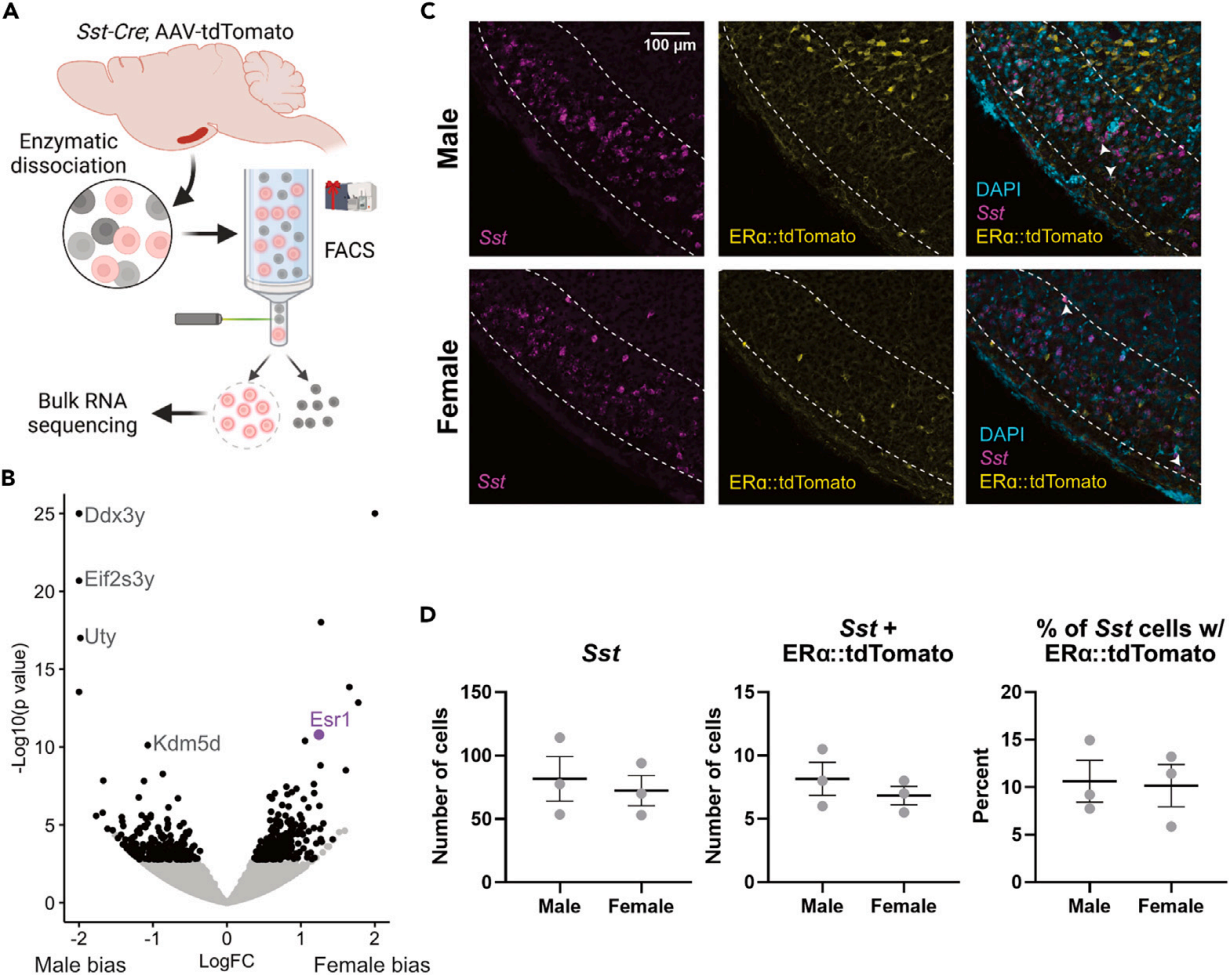
A subset of TN^SST^ neurons is sensitive to estrogens (A) Schematic of Flow-Seq analysis (also applies to Figure 5 genetic input). AAV-flex-tdTomato was stereotaxically injected into the TN of *Sst-Cre* mice. Lateral hypothalamic region was grossly dissected and enzymatically dissociated before segregating *Sst*^*+*^ red neurons via flow cytometry. Resultant cells were sent for bulk RNA sequencing. Created using BioRender.com. (B) Numerous genes are more differentially expressed between females and males (black dots), including canonical Y-associated genes (*Ddx2y*, *Eif2s3y*, *Uty*, and *Kdm5d*; gray text) and *Esr1* (purple dot). (C) Representative fluorescent images from *Esr1-Cre* mice showing colocalization of *Sst* (magenta), *Esr1::tdTomato* (yellow), and DAPI nuclear stain (cyan). White arrows indicate *Sst-Esr1* double-labeled cells. Scale bar = 100 μm. (D) Quantification of colocalization confirms *Esr1*/*Sst* co-expression but reveals no sex difference. Mean ± SEM.

To test if TN^SST^ neurons, specifically, can respond to estrogens and/or signals from white adipose tissue, we profiled the transcriptome of fluorescently labeled TN^SST^ neurons using flow cytometry followed by bulk RNA sequencing (Flow-Seq; Figure 4A). Transcriptomic analysis of isolated TN^SST^ neurons uncovered numerous differentially expressed genes between females and males, with the gene for estrogen receptor alpha, *Esr1*, being more highly expressed in females (Wχ^2^ = 6.736, adj p = 2.356 × 10^−8^; Figure 4B). However, we were unable to detect ERα immunoreactivity in the TN using standard antibodies (Millipore Sigma 06-935). Instead, we confirmed expression from the *Esr1* locus through the injection of a Cre-dependent tdTomato reporter into the TN of female and male *Esr1-Cre* mice. Subsequent colocalization of tdTomato with *Sst* via *in situ* hybridization revealed co-expression of *Esr1* in approximately 10% *Sst*-expressing cells across sexes (Figures 4C and 4D), confirming that at least a subset of TN^SST^ neurons is sensitive to estrogens.

**Figure 5:**
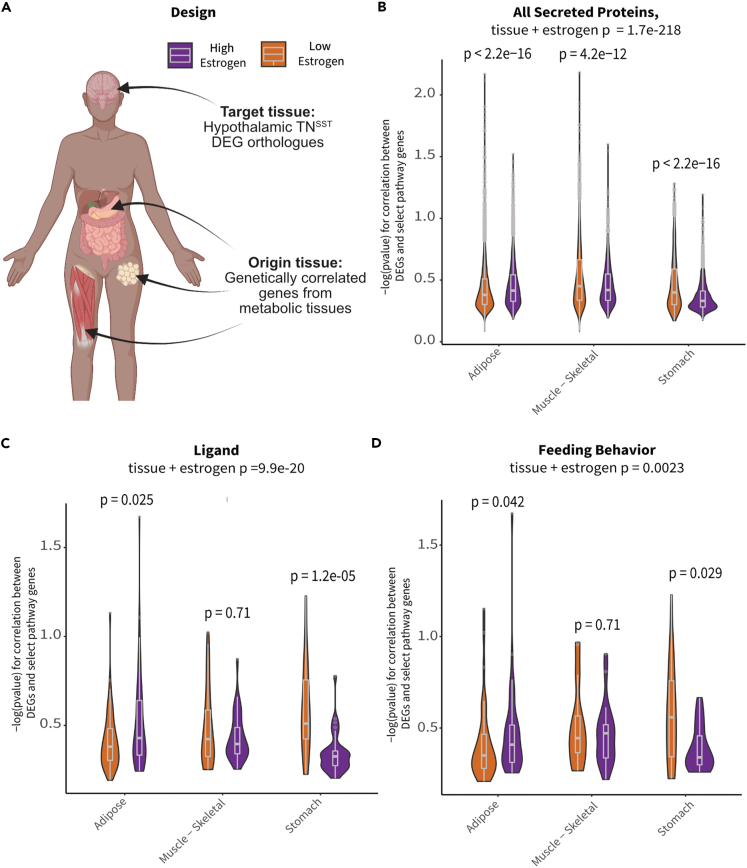
TN^SST^ neurons display increased hormonal pathway co-correlations with white adipose tissue in individuals with inferred high estrogen signaling (A) Schematic overview of co-correlation analysis. High-expressing TN^SST^ genes from mouse Flow-Seq experiments were co-correlated across various peripheral metabolic tissues across high- and low-estradiol groups identified in the GTEx database. Created with BioRender.com. (B) Inferred estradiol levels affected co-correlation pathways relevant to all secreted proteins. High-estradiol individuals showed increased co-correlations in adipose tissue whereas those with lower estradiol showed increases in skeletal muscle and stomach communication. (C) Similar trends in adipose and stomach co-correlations across estradiol groupings were seen for ligand pathways. (D) Co-correlations across tissues showed differential impact on genes associated with feeding pathways. Individuals with higher estradiol showed increased co-correlations within these pathways with adipose tissue and decreased co-correlations with stomach as compared to those with lower estradiol levels. (B)–(D) depict violin density plots with superimposed box-and-whisker plots denoting medium, first and third quartiles, and minimum and maximum values. Non-parametric ANOVA and post hoc t test p values reported on graphs.

To determine if TN^SST^ neurons communicate with adipose tissue or vice versa, we used a co-correlation analysis method based on genetic variation. High-expressing genes from TN^SST^ neurons (based on counts > glial fibrillary acidic protein expression) were used as the “target” pathways for human orthologs within the GTEx database^60^ and subjected to cross-tissue genetic co-correlational analyses^61^^,^^62^ (Figure 5A). As previous data indicated that TN^SST^ neuron responsivity to metabolic cues might be localized to periods of higher circulating estradiol (Figure 2D) and TN^SST^ may be able to directly sense circulating estradiol levels (Figure 4), individuals in the GTEx database were binned into groups with either “high” or “low” circulating estradiol levels via weighted aggregation of pan-tissue *Z* scores corresponding to estrogen-responsive gene expression (Figures S3A–S3D). Binning individuals into groups with indicators of “low” or “high” estrogen signaling revealed weakly associated groups (as per biweight midcorrelation^63^; bicor coefficient = −0.32, p = 0.0023), suggesting that strong cross-tissue interactions differed depending on estrogen signaling status. Next, genetic co-correlation analyses from adipose (subcutaneous & omental), skeletal muscle, stomach, and small intestine to hypothalamic highly expressed genes were conducted. A lack of significant co-correlations with small intestine and several other tissues resulted in these tissues being omitted from the rest of analyses. A contributing factor for this lack of significance may be the limited number of individuals with matching tissue expression (Figure S3E). Given that subsets of strong cross-tissue correlations remained between the other tissues and highly expressed hypothalamic gene orthologs, relevant pathways which might contribute to signaling were examined accordingly (Figures 5B–5D). Significant interactions for co-correlations between estrogen signaling group and tissue were observed across tissues for all secreted proteins (Kruskal-Wallis test for interaction between estrogen category + tissue p = 1.7 × 10^−218^; Figure 5B), known ligands (Kruskal-Wallis interaction p = 9.9 × 10^−20^; Figure 5C), peptide hormones (Kruskal-Wallis interaction p = 1.8 × 10^−13^, data not shown), and feeding behavior pathways (Kruskal-Wallis interaction p = 0.0023; Figure 5D). In addition, several pathways showed specificity in strength of co-correlations from one tissue to another. For example, individuals in the higher inferred estrogen signaling group exhibited higher co-correlations between TN^SST^ and adipose within secreted proteins (p < 2.2 × 10^−16^), ligand (p = 0.025), and feeding behavior pathways (p = 0.042) as compared to individuals in the low-estrogen signaling group. Interestingly, this relationship was reversed for all secreted proteins in skeletal muscle, with individuals in the low-estrogen signaling group exhibiting higher co-correlations (p = 4.2 × 10^−12^). These results indicate increased communication between adipose and TN^SST^ neurons during periods of high estrogen signaling, and a switch to skeletal muscle communication when estrogen signaling is low. Across all gene sets, individuals with inferred low estrogen signaling exhibited higher co-correlations with stomach as compared to individuals with high estrogen signaling (all secreted proteins: p < 2.2 × 10^−16^; ligands: p = 1.2 × 10^−5^; peptide hormones: p = 1.8 × 10^−4^; and feeding behavior: p = 0.029). Together, these human genetic co-correlation data indicate that TN^SST^ neurons modulate feeding pathways through preferential communication with adipose when estrogen signaling is high and stomach hormones when estrogen signaling is decreased. These observations are in line with known responsivity of TN^SST^ neurons to stomach peptide hormone and known regulator of feeding ghrelin,^43^ but indicate that this communication pathway may be more salient when estradiol levels or estrogen signaling is low. Further, these analyses of human data may suggest a similar integration of metabolic cues alongside reproductive hormones in humans as well as mice.

**Figure 6:**
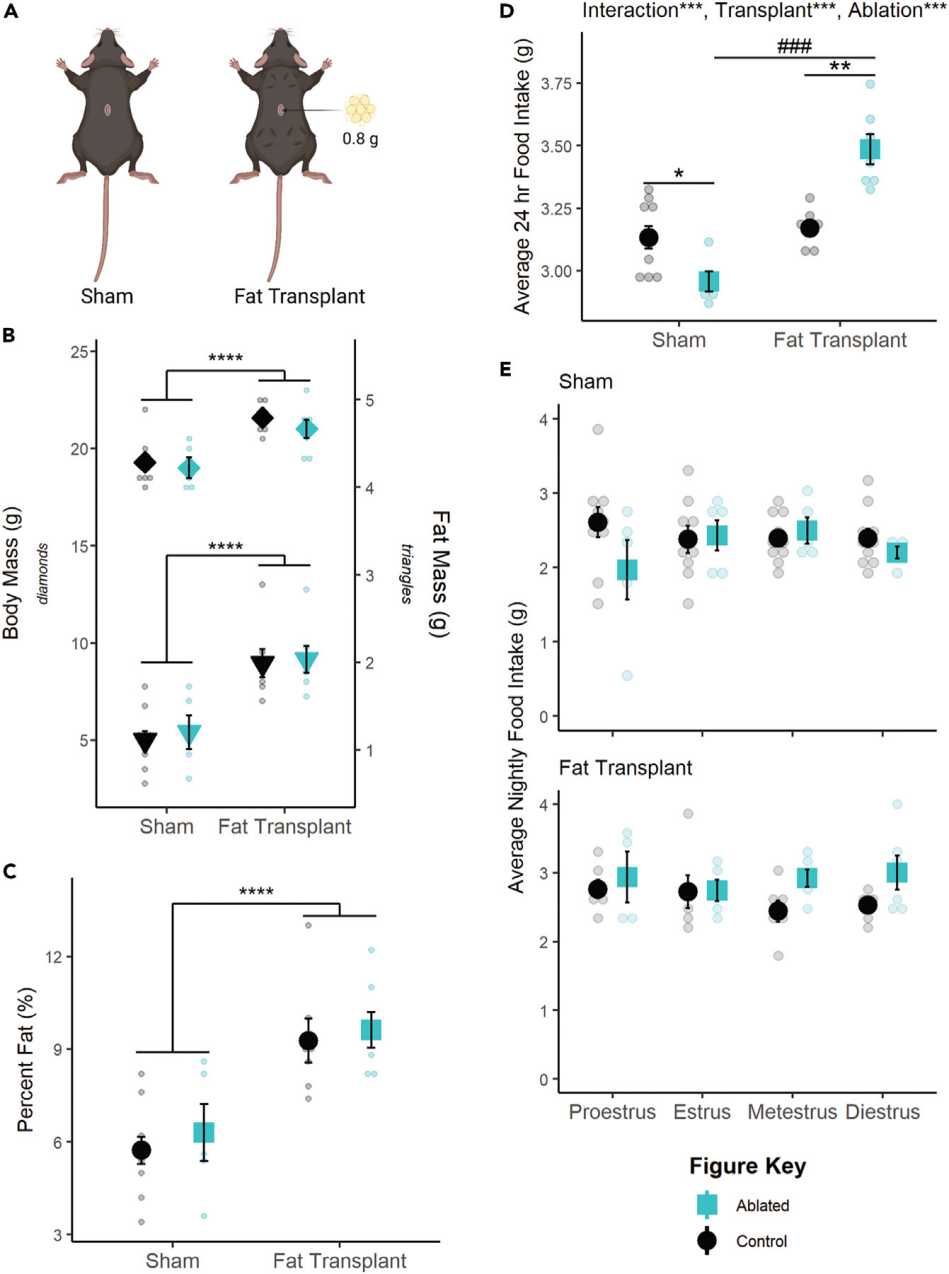
Fat transplantation modulates the effect of SST neuron ablation (A) Representative schematic of fat transplants. Four deposits of 0.2 g each were placed in the dorsal subcutaneous region. Created with BioRender.com. (B) Fat transplant increases raw body mass (left axis, p < 0.0001) and fat mass (right axis, p < 0.001) regardless of SST neuronal ablation. (C) This translates to an overall increase in adiposity (p < 0.0001). (D) Fat transplantation reverses the effect of TN^SST^ ablation, significantly increasing daily food intake compared to non-ablated controls. (E) This effect of fat transplant (top panel) seems to be due to a lack of effect during proestrus, though data were underpowered to detect the effect of estrous stage in sham controls (bottom panel). Mean ± SEM; ANOVA and post hoc t tests where applicable; within adiposity group: ∗p < 0.05, ∗∗p < 0.01; ∗∗∗p < 0.001, ∗∗∗∗p < 0.0001; between adiposity group: ^###^p < 0.001. Sham: Control n = 10, Ablated n = 5; Transplant: Control n = 7, Ablated n = 7. Sham: Proestrus Control n = 10 & Ablated n = 5, Estrus Control n = 9 & Ablated n = 5, Metestrus Control n = 10 & Ablated n = 5, Diestrus Control n = 10 & Ablated n = 4; Fat Transplant: Proestrus Control n = 6 & Ablated n = 5, Estrus Control n = 6 & Ablated n = 6, Metestrus Control n = 6 & Ablated n = 6, Diestrus Control n = 6 & Ablated n = 6.

To test the causal, directional relationship between fat and tuberal hypothalamic SST neurons in the modulation of food intake, caspase ablation studies were repeated in combination with fat transplantation. Approximately 1.5 weeks following transplantation of ∼0.8 g subcutaneous fat (Figure 6A), recipient mice exhibited significantly increased body mass (F(1,25) = 28.3184, p < 0.0001; Figure 6B), raw fat mass (F(1,25) = 34.2342, p < 0.0001; Figure 6B), and percent fat mass (F(1,25) = 30.0008, p < 0.0001; Figure 6C) and no interaction with SST neuronal ablation in any case. Thus, fat transplantation increased adiposity similarly across neuronal ablation groups.

Fat transplant also increased food intake in general (F(1,25) = 52.524, p < 0.0001), and the effect of SST neuronal ablation was affected by fat transplant (F(1,25) = 26.660, p < 0.0001; Figure 6D). Post hoc t tests revealed that SST neuronal ablation significantly decreased food intake in sham transplant animals (t(11.646) = -2.917, p = 0.01327) but significantly increased food intake in animals receiving fat transplant (t(4.8.2536) = 4.8427, p = 0.001175), similar to the previous relationship with body mass in proestrus (Figure 3A). However, we were unable to detect a significant interaction with fat transplantation and ablation status over the estrous cycle (Figure 6E), possibly due to high variability in nighttime food intake in sham-transplanted mice. Together, these findings indicate that fat transplantation masks the effect of SST neuron ablation and reveal a role for fat mass in modulating the function of tuberal hypothalamic SST neurons within the feeding circuit.

## Discussion

These data suggest that tuberal hypothalamic SST neurons are a locus in the brain that mediates metabolic and reproductive crosstalk. While activation of TN^SST^ neurons increases food intake across sexes, permanent inactivation of ARC^SST^ and TN^SST^ neurons by ablation during adulthood results in decreased food intake only in females during the proestrus phase. This effect depends on body mass, as it is apparent only in lighter animals. In wild-type mice, body mass inversely correlates with food intake on the night of proestrus, but SST neuron ablation uncouples this relationship. Further analysis reveals that white adipose tissue abundance is a significant contributing factor. Not only does postmortem adipocyte size correlate with body mass in neuron ablation experiments, but also fat transplantation studies confirm that SST neuron ablation only decreases food intake in lean animals compared to their fat-transplanted counterparts. An interaction between cycling adipokines and gonadal hormones may be mediated by the direct effects of these circulating molecules on SST neurons, as these cells show some estrogen sensitivity via co-expression analyses. Furthermore, co-correlations between the hypothalamus and adipose tissue in humans and fat transplantation experiments in mice point to the importance of secreted proteins and ligands, suggesting SST neurons may detect and respond to adipokines. Future studies are needed to confirm and dissect the mechanisms of these cellular effects.

What adipokine factor is possibly being detected by the SST neurons (and specifically by the TN^SST^ population) remains to be determined. Leptin positively correlates with overall adiposity,^64^ and it has been known to play a crucial role in reproductive responsiveness to metabolic condition, namely as the permissive signal required for pubertal onset.^65^^,^^66^^,^^67^ Adiponectin, an adipokine that negatively correlates with visceral fat mass in mammals,^64^ has long been shown to downregulate reproduction through direct impacts on the hypothalamus.^68^ Resistin is correlated with higher overall adiposity,^69^ exhibits numerous interactions with the hypothalamic-pituitary-gonadal axis,^70^^,^^71^^,^^72^ and is downregulated during the fertile periods of the mouse estrous cycle.^73^

Regardless of adipokine contributor, this crosstalk paradigm provides a plausible explanation for the varied effects of estradiol on food intake in mice. While endogenous fluctuations and experimental manipulations of estradiol consistently reveal that estrogens decrease food intake in rats and guinea pigs,^74^^,^^75^^,^^76^^,^^77^ the mouse literature is less definitive.^76^^,^^78^^,^^79^^,^^80^^,^^81^^,^^82^^,^^83^ Indeed, in our hands, ovariectomy decreased food intake across ablation groups, as has been seen previously in mice.^81^^,^^83^ Instead, the more consistent phenotype in mice is a decrease in energy expenditure following estradiol depletion.^10^^,^^12^^,^^13^^,^^83^ In light of this study, it is possible that the effects of estradiol on feeding across mouse studies, as observed by either endogenous estrous cycle fluctuations or ovariectomy manipulation, could be confounded by body mass and adiposity. Thus, factors like age at time of experiment, differences in fat distributions between species or strains, diet, or ovariectomy, and time from ovariectomy to estradiol replacement might present confounds based on changes to fat and/or lean mass.

How circulating estrogen levels contribute to this circuit also requires further investigation. Our human GTEx analyses show that co-correlations between genes expressed in TN^SST^ and that in peripheral tissue shift from predominantly adipose-based in high estrogen conditions to skeletal muscle- and stomach-based in low estrogen signaling conditions (Figure 5). This suggests that higher estrogen levels may increase communication between TN^SST^ neurons and white adipocyte depots, particularly as it relates to regulation of feeding behavior (Figure 5D). While this could be due to the actions of circulating estrogens on white adipose tissue itself (reviewed by Palmer and Clegg and Hevener et al.^1^^,^^84^), it is also possible that estrogens directly act on TN^SST^ (and/or ARC^SST^) neurons to increase their sensitivity and/or responsivity to adipokines. Indeed, a subset of TN^SST^ neurons exhibit estrogen sensitivity (Figure 4), and ARC^SST^ neurons have been found to be estrogen sensitive^47^ and responsive^48^ in non-rodent models. Future studies would be needed to test for a possible direct effect on either of these populations in mice. Previous studies of the TN include both female and male mice but do not report *Esr1* expression or estrogen sensitivity in *Sst*+ neurons.^42^^,^^43^ However, the idea that estrogen receptor is expressed in the TN and that the function of TN neurons is sensitive to estrous cycle stage is consistent with spatial mapping of *Esr1*^+^ neurons to a continuous region that spans the ventrolateral region of the ventromedial hypothalamus and the TN.^85^ It is also plausible that the concomitant decrease in effective communication with the stomach in high-estrogen signaling states is due to an interaction of estradiol with the hunger hormone ghrelin. TN^SST^ neurons are directly responsive to ghrelin,^43^ while estradiol can blunt the orexigenic effects of ghrelin via reduced ghrelin treatment efficacy^77^ and reduced release of the hormone.^86^ Thus, estradiol may indirectly influence TN^SST^ activity and subsequent food intake via regulation of ghrelin release and/or signaling.

Estradiol might also be detected elsewhere in the brain and impact TN^SST^ and ARC^SST^ neuronal modulation of feeding through integration at the circuit level. TN^SST^ neurons project to many estrogen-sensitive nodes or nodes receiving direct input from estrogen-responsive regions, including the bed nucleus of the stria terminalis, parabrachial nucleus, and central amygdala.^43^ Importantly, bifurcated TN projections to the paraventricular nucleus and bed nucleus of the stria terminalis have been shown to mediate the feeding effects of TN^SST^ neurons.^43^ As a known target for arcuate AgRP neurons—cells which have been shown to integrate reproductive and metabolic cues^31^—the paraventricular nucleus may contribute to the integration of metabolic and reproductive cues as a downstream integrative node. However, given that ablation of TN^SST^ neurons eliminated the changes in the relationship between body weight and food intake over the estrous cycle, it is plausible that nodes of estradiol detection are TN^SST^ neurons and/or neurons upstream of the TN. This circuit-wide integration of estradiol is a known mechanism of action for the gonadal hormone, with estrogens acting on many circuit nodes to coordinate behavioral output in a variety of cases, including reward/addiction^87^ and thermoregulation.^88^ It is therefore probable that the effects of estradiol on feeding function similarly, as the anorexigenic effects of estradiol have been localized to numerous feeding nodes such as the hypothalamic arcuate nucleus^89^^,^^90^^,^^91^^,^^92^^,^^93^ and the nucleus of the solitary tract of the brainstem.^94^^,^^95^ Retrograde tracing studies would help determine if these or estrogen-sensing regions (including the ventral subiculum, an upstream region targeting TN^SST^ neurons^44^ known to express estrogen receptors^96^^,^^97^) are candidate nodes mediating the effects of estradiol seen in this study.

Alternatively, ARC^SST^ neurons may detect fluctuating hormone levels to modulate the observed phenotype. In ewes, a subset of ARC^SST^ neurons colocalize with *ESR1* and increase their activity in response to estradiol treatment.^47^ Should this pattern of activation be confirmed in mice, it would suggest a mechanism whereby activation of ARC^SST^ neurons during high periods of estradiol induces feeding under low adiposity conditions.

In all, this study adds to the growing literature interrogating the contributions of TN^SST^ neurons to feeding behavior. Central SST (originally named growth hormone inhibiting hormone in the central nervous system)^98^ had long been known to affect food intake through somatostatin receptor 2.^33^^,^^37^^,^^38^^,^^39^^,^^40^^,^^41^^,^^45^^,^^99^^,^^100^^,^^101^^,^^102^ This effect was seemingly localized to the lateral tuberal nucleus, where TN^SST^ neurons were found to integrate into the melanocortin feeding system, though the effect of these neurons on feeding was attributed to γ-aminobutyric acid (GABA) release as opposed to direct SST effects.^43^ However, given that dynamics of GABA, NPY, and AgRP release from arcuate neurons in the temporal regulation of food intake,^103^ it is possible that similar dynamics are apparent between GABA and SST in the TN. In line with previous studies,^42^^,^^43^ chemogenetic activation of TN^SST^ increased food intake across animals. Lack of excess crumbs in subsequent caspase ablation experiments confirmed that this is not a side effect of gnawing; a possibility also excluded in a previous study looking at wood stick gnawing.^43^ However, a subsequent study did see an increase in gnawing behavior with chemogenetic activation, probably due to a larger target area encompassing large portions of the lateral hypothalamus.^42^ Furthermore, whereas caspase ablation did decrease food intake, previous studies pooled across the sexes.^43^ Similar pooling in our hands also resulted in an overall decrease in food intake with SST neuron ablation, indicating that females possibly carry the effect when there is low power to detect sex differences. Alternatively, the inclusion of ARC^SST^ neurons in our ablation may have also resulted in the observed sex difference and integrative capacities of tuberal hypothalamic SST neurons at large.

In our hands, this ablation-mediated decrease in food intake did not result in any alteration to body weight. This is in line with a previous study demonstrating that while the decrease in food intake may be due to ablated animals gaining weight more slowly, there are no raw body weight differences that result.^43^ Previous studies have also been conflicted on the role of TN^SST^ neurons on movement and locomotion. While one study demonstrated a decrease in total locomotion and increase in vertical movement with TN^SST^ neuron ablation,^43^ another study found no change in walking and showed an increase in vertical movement with TN^SST^ neuron activation.^42^ Our telemetry results suggest a lack of change in movement in general with SST neuron ablation. The differences among these studies may be due to the methods of movement quantification and/or viral spread of ablation. Similarly, while our study did not measure drinking behavior, it is unclear whether and how TN^SST^ neuron ablation is involved in thirst processes, with one study demonstrating the necessity of TN^SST^ neurons to maintain baseline drinking levels^43^ but another failing to show TN^SST^ neuron activation as sufficient to induce increases in drinking.^42^

TN^SST^ neurons were also found to contribute to food context learning in males (N.B.: in all external papers discussed, no definitions for sex category were ever provided. In mice, we assume that sexes were defined using anogenital distance.),^44^ indicating that the TN may straddle homeostatic and hedonic feeding mechanisms.^14^ And although many of these previous studies include female and male mice, they do not report *Esr1* expression or estrogen sensitivity in TN^SST^ neurons. Thus, the current paper adds to this growing literature by not only delineating an apparent sex difference but also context dependence in tuberal hypothalamic SST neuronal modulation of food intake.

We further speculate that tuberal hypothalamic SST neurons serve as a nexus of integration and a mediator of reproductive and metabolic interactions within the feeding circuit. In cycling rodents, fertile periods during the estrous cycle are accompanied by alterations to metabolic output, including a decrease in food intake,^74^^,^^75^^,^^76^^,^^104^ increase in locomotion,^104^^,^^105^^,^^106^^,^^107^ and increased core body temperature.^105^^,^^107^ These changes are hypothesized to suppress energy intake and promote active mate-seeking behavior and sexual receptivity. This study identifies tuberal hypothalamic SST neurons as possible mediators of such interactions, actively promoting energy intake during fertile periods when metabolic reserves may be insufficient to support reproduction.

### Limitations of the study

Viral spread implicates both TN^SST^ and ARC^SST^ neurons in the conditional modulation of food intake. However, targeting toward TN^SST^ neurons was more complete and robust across studies. Future studies dissecting the specific contributions of these two regions would greatly add to circuit delineation and perhaps illuminate the specific locations where metabolic and/or reproductive cues modulate circuitry function.

Additionally, while the integration of reproductive and metabolic states makes adaptive sense to occur in gestating mammals where metabolic requirements are vital to reproductive success, it is formally possible that this same mechanism could be engaged in non-gestating mammals (in this case, males) in certain contexts. For example, the effect might be masked in males due to their increased weight and adiposity at the time of testing at 8 weeks of age in this study. Future research could utilize mice with low adiposity to determine if the integrative capacity of the feeding circuit extends across sex.

Finally, while our approach to stratifying the GTEx database was creative and aligns with known data about deceased individuals therein, the estimations of circulating estrogens cannot be confirmed without either additional information regarding hormonal treatment, reproductive status, and/or salivary or blood serum hormone levels. Databases should consider adding additional information to their collections where financially feasible to support more nuanced analyses of variables related to sex and reproductive state.

## STAR★Methods

### Key resources table

**Table:** 

REAGENT or RESOURCE	SOURCE	IDENTIFIER
**Antibodies**		

rabbit anti-cFOS	Synaptic Systems	Cat#226003; RRID: 2231974
goat anti-rabbit Alexa Fluor 488	Thermo Fisher	Cat#A11034; RRID: AB_2576217
rabbit anti-DsRed	Takara Bio Clontech	Cat#632496; RRID: AB_10013483

**Bacterial and virus strains**		

AAV2-flex-taCasp3-TEVp	UNC Vector Core (Depositor: Nirao Shah & Jim Wells) Yang et al.^58^	
AAV8-hSyn-hM3D(Gq)-mCherry	Addgene (Depositor: Brian Roth) Krashes et al.^57^	Addgene viral prep Cat#50474-AAV8
AAV2-FLEX-tdTomato	Addgene (Depositor: Edward Boyden)	Addgene plasmid Cat#28306
AAV8-Syn-FLEX-Mac-GFP	Addgene (Depositor: Edward Boyden)	Addgene plasmid Cat#58852

**Chemicals, peptides, and recombinant proteins**		

Clozapine-n-oxide (CNO)	Millipore Sigma	Cat#0832
Giemsa	Fisher	Cat#G146-10
DIG RNA labeling kit	Roche	SKU#11277073910
Fluorescein RNA Labeling Mix	Roche	SKU#11685619910
RNA Clean & Concentrator	Zymo Research	Cat#R1017
ZymoPURE II Plasmid Midiprep kit	Zymo Research	Cat#D4201
Zymo DNA Clean & Concentrator	Zymo Research	Cat#D4033
Blocking Reagent	Roche	Cat#11096176001
TSA Plus Cyanine 5 System	Akoya Biosciences	Cat#NEL745001KT
Rneasy Micro kit	Qiagen	Cat#74004

**Deposited data**		

RNA seq data from neurons FACS isolated from mouse tuberal hypothalamus neurons	NCI Gene Expression Omnibus (GEO)	GSE224254
GTEx	Broad Institute	Version 8
Molecular Signatures Database	Broad Institute	Human Gene Set: HALLMARK_ESTROGEN_RESPONSE_EARLY
Datasets for estrogen signaling binning of human data	Github	https://github.com/Leandromvelez/sex-specific-endocrine-signals.

**Experimental models: Organisms/strains**		

model organism: *Sst-Cre*: genotype STOCK *Sst*^*tm2.1(cre)Zjh*^/J	The Jackson Laboratory	Strain#013044
model organism: *Esr1-Cre*: genotype B6N.129S6(Cg)-*Esr1*^*tm1.1(cre)And*^*/J*	The Jackson Laboratory	Strain#017911

**Recombinant DNA**		

cDNA for *Sst* (bases 7-550 for transcript variant 1, bases 317-860 for transcript variant 2)	Allen Brain Atlas	Probe: RP_Baylor_103041
TA Cloning™ Kit, with pCR™2.1 Vector and One Shot™ TOP10F' Chemically Competent *E. coli*	Invitrogen	Cat#K203001

**Software and algorithms**		

VitalView Software	Starr Life Sciences	version 5.1
lme() function (nlme package, version 3.1-157)	r-project.org	R version 4.2.1
t_test() function (rstatix package, version 0.7.0)	r-project.org	R version 4.2.1
Code for volcano plot	Github	http://github.com/jevanveen/ratplots R function: deseq_volcano_plot_gs()
Analysis pipleline for estrogen signaling binning of human data and cross-tissue correlations with mouse orthologues	Github	https://github.com/Leandromvelez/sex-specific-endocrine-signals
Kallisto	Pachter Lab Bray et al.^114^	version 0.46.2
Deseq2	Galaxy	Version 2.11.40.6+galaxy1
CellProfiler	Broad Institute	4.2.1

### Resource availability

#### Lead contact

Further information and Requests for resources and reagents should be direct to and will be fulfilled by the lead contact, Stephanie Correa (stephaniecorrea@ucla.edu).

#### Materials availability

The study did not generate new unique reagents.

### Experimental model and study participant details

#### Mice

Female (defined as having small anogenital distance at weaning and presence of ovaries at time of death) and male (defined as having large anogenital distance at weaning and presence of testes postmortem) mice expressing the *Sst-Cre* driver transgene (JAX stock no. 013044, *Sst*^*tm2.1(cre)Zjh*^/J) were maintained on a C57BL/6J genetic background. Heterozygotes (*Sst-Cre/+*) and/or wildtype littermates (*+/+*) were used for all studies. Genotypes were determined as per JAX protocol 28317. Female and male mice expressing the *Esr1-Cre* driver transgene (JAX stock no. 017911, B6N.129S6(Cg)-*Esr1*^*tm1.1(cre)And*^*/J*) were maintained on a C57BL/6J genetic background. Heterozygotes (*Esr1-Cre/+*) were used for colocalization studies. Genotypes were determined as per primers from JAX protocol 27213. Experiments were performed on cycling females and gonadally-intact males unless otherwise stated. Mice were maintained on a 12:12 light cycle, with *ad libitum* access to food and water (unless otherwise specified), under controlled humidity conditions at 22-23C, and in single-housed cages with non-caloric paper bedding to ensure accurate food intake assessment. All studies were carried out in accordance with the recommendations in the Guide for the Care and Use of Laboratory Animals of the National Institutes of Health. UCLA is AALAS accredited, and the UCLA Institutional Animal Care and Use Committee (IACUC) approved all animal procedures.

### Method details

#### Estrous cycle staging

Vaginal lavages were performed on females daily, between ZT 0 and ZT 4, using 30 μL of standard phosphate buffered saline (PBS). Samples were deposited onto slides and allowed to dry prior to staining. Males were subjected to similar handling during this time to ensure roughly equivalent handling stress. Giemsa staining was carried out to visualize cellular composition of the vaginal cavity. Stock Giemsa stain was prepared at least one week in advance of use. An 18.5% solution of Giemsa powder (Fisher G146-10) in glycerin was heated to 60°C and cooled before diluting 9:14 with 100% methanol. Stock was diluted 1:30 in PBS before use, shaking vigorously before stain. Slides were incubated for one hour at room temperature. Prior to staining, slides were briefly fixed in 100% methanol. Staging was assessed via light microscopy to determine the relative abundance of leukocytes, nucleated epithelial cells, and cornified epithelial cells^108^ and stages were assigned with morning samples indicating the prior night’s estrous stage, to align the cytology stage to the behavioral changes that occur in the dark (night) of estrus.^109^ This staging method was confirmed by examining patterns of core body temperature during the dark phase across the estrous cycle, which also changes during estrus.^105^

#### Surgical procedures

Mice received analgesics (0.074 mg/kg buprenorphine two times daily, 7.11 mg/kg carprofen one time daily) on the day of and one day post-surgery. Mice were anaesthetized with 3% isoflurane and maintained within a range of 1.25-2.5%. AAVs were bilaterally injected into the TN of adult mice (coordinates relative to Bregma: A-P -1.65 mm, lateral ±0.75, D-V -5.45; scaled when Bregma-Lambda distance was not equivalent to 4.2 mm) at a rate of 5 nL/s using a glass-pulled needle. See below table for titers and injection volumes. Controls consisted of both wildtype animals injected with the experimental virus (virus controls) and Cre positive animals injected with cell-filling GFP (genotype controls). Ovariectomy surgeries included complete removal of gonads from adult mice. Gonadectomies occurred immediately prior to stereotaxic viral injections within the same surgical period. In telemetry experiments, G2 eMitters (Starr Life Sciences) were implanted intraperitoneally on the same day as viral injection. Experiments were conducted following at least two weeks recovery from surgical proceedings.

**Table:** List of viral vectors used

Experiment	Virus	Depositor & procurement	Titer (vg/mL)	Volume (nL)	Citation
Caspase ablation	AAV2-flex-taCasp3-TEVp	Nirao Shah & Jim Wells, UNC Vector Core	1–8 x 10^12^	200–250	Yang et al.^58^
Transient activation	AAV8-hSyn-hM3D(Gq)-mCherry	Brian Roth, Addgene viral prep # 50474-AAV8	≥4 × 10^12^	150–200	Krashes et al.^57^
Fluorescent localization	AAV2-FLEX-tdTomato	Edward Boyden, Addgene plasmid #28306	≥5 × 10^12^	200 for Flow-Seq; 400 for *Esr1-Cre*	
Fluorescent controls	AAV8-Syn-FLEX-Mac-GFP	Edward Boyden, Addgene plasmid #58852	1:5 dilution of stock	Matching volume to experimental animals	

#### Caspase ablation experiments

Gross movement and core body temperature were passively measured every other week for eight weeks using VitalView software (Starr Life Sciences). Body weight was measured every week. Food assay was performed when mice were not on telemetry pads. At ZT 0.5 on the start day of the experiment, 2/3 of the non-caloric paper bedding was removed. A pre-measured amount of food was delivered, and mouse body weight measured. Food in hopper was weighed at ZT 0.5 and ZT 11.5 every day until experiment conclusion. After 96 hours, food and all bedding, including interspersed crumbs, were collected. Measurements for total food were obtained by subtracting the final weight of food pellets and crumbs from the original weight of food provided. Measurements for crumbs were obtained by subtracting the final weight of food pellets and crumbs from the final weight of food in the cage hopper. Both measurements were divided by 4 to obtain the average food eaten or crumbs produced per day (24 h food intake). For estrous cycle experiments, 13-hour nightly food intake (ZT 11.5 – ZT 0.5) was utilized instead as we were more confident in nightly staging based on the behavioral changes that occur during the night of estrus^109^ and our ability to confirm estrus staging with core body temperature in the dark phase of estrus.^105^ For some experiments, 4-5 hour fasted glucose tolerance tests were performed prior to sacrifice. In ovariectomy experiments, two food assays were performed back-to-back, non-fasted resting glucose levels were collected, body composition was measured via NMR, and indirect calorimetry was performed in Oxymax metabolic chambers (Columbus Instruments) at room temperature. Upon experiment completion, all brains were collected using RNase-free conditions. Inguinal white adipose tissue (iWAT) and gonadal white adipose tissue (gWAT) were collected for histology analyses.

#### Transient activation food intake assay

Clozapine-n-oxide (CNO; MilliporeSigma #0832) was used to activate TN^SST^ neurons in *Sst-Cre* animals expressing hM3Dq-mCherry. Stock solution of 20 mg/mL in DMSO was stored at -20°C and diluted to a working solution of 0.03 mg/mL in sterile saline also stored at -20°C. Saline control (0.15% DMSO) or CNO (10 μL/g body weight, dose of 0.3 mg/kg) working solution were administered IP in a counterbalanced design. Experiments were completed in duplicate replicate trials. Mice were transferred to experimental room at least 15 minutes prior to experimentation. Experiments were begun between ZT 2-3 and terminated between ZT 6-7. Following injection, food intake was measured at 0.5, 1, 2, and 4 hr. Vaginal lavage was performed on female mice after experiment conclusion to prevent stress interference with food intake. All mice were injected with CNO 90 minutes prior to sacrifice to enable neuronal activation validation via cFOS immunohistochemistry.

#### Fat transplantations

Donor fat was taken from various visceral (i.e., periuterine perigonadal, retroperitoneal, and omental) depots of wildtype female C57BL/6J mice and implanted into female mice recently stereotaxically injected under standard surgical conditions. Four depots of 0.15-0.25g were placed subcutaneously on the dorsal surface through a single incision mid-back, for a final transplantation total of 0.6-0.9g of white adipose. Fat for each depot was divided into at least three individual pieces to promote vascularization. The visceral-to-subcutaneous paradigm was used due to the deleterious metabolic effects of this graft.^110^ Food intake was assayed 2-3 weeks following transplantation to allow for sufficient angiogenesis^111^ and graft stabilization without endogenous fat depot compensation.^112^ Upon sacrifice, grafts were examined to confirm tissue was not necrotic.

#### Histology

##### *In situ* hybridization (ISH) and immunostaining (IHC)

*Sst* sense and antisense probes were transcribed using a DIG or FITC RNA labeling kit (Roche) and purified with RNA Clean & Concentrator (Zymo Research). PCR products were amplified using Allen Brain Institute-derived reference primer sequences and cloned into pCR 2.1 TOPO (Invitrogen). Plasmid DNA was then isolated from bacterial cultures (ZymoPURE II Plasmid Midiprep kit), linearized, and purified (Zymo DNA Clean & Concentrator). Validation of caspase ablation was carried out on 35μm-thick coronal slices via chromogen ISH using BCIP (5-bromo-4-chloro-3-indoyl phosphate) and INT (iodonitrotetrazolium). Validation of hM3Dq targeting and activation was accomplished by visualization of native mCherry expression and IHC stain for cFOS. Briefly, slides were blocked and incubated with rabbit anti-cFOS (1:200, Synaptic Systems # 226003, RRID: 2231974) primary antibody overnight at 4°C. The next day, sections were incubated for 1 hour at room temperature with goat anti-rabbit Alexa Fluor 488 secondary (1:500, Thermo Fisher Scientific # A11034, RRID: AB_2576217) and counterstained with DAPI. For colocalization experiments, *Esr1-Cre* mice were bilaterally injected with 400 μl AAV2-flex-tdTomato into the TN coordinates. Native tdTomato fluorescence destroyed by combined ISH protocol was recovered by rabbit anti-DsRed (1:1000, Takara Bio Clontech # 632496, RRID: AB_10013483) antibody and switched to the green channel using an Alexa Fluor 488 secondary. Dual *Sst* ISH & tdTomato IHC protocol was accomplished via TSA amplification. Briefly, 35 μm sections were fixed, permeabilized with Triton X-100, and acetylated before overnight ISH probe incubation at 65°C. The next day, tissue was then washed, blocked with Blocking Reagent (MilliporeSigma 11096176001Roche) and heat inactivated sheep serum, and incubated with anti-DsRed overnight at 4°C. The final day, tissue was washed before ISH signal was developed with the TSA Plus Cyanine 5 System (Akoya Biosciences # NEL745001KT). Slides were then stripped of horseradish peroxidase and blocked with normal goat serum before incubating with goat anti-rabbit Alexa Fluor 488 (1:400) for 2 hours at room temperature.

##### DREADD viral injection mapping

Sections were anatomically matched using the Allen Mouse Brain Atlas (mouse.brain-map.org), and the anatomical areas with hM3Dq-mCherry positive cells were manually identified and outlined in Illustrator. Areas containing dense hM3Dq-mCherry expression were outlined with 5% opacity, and areas containing sparse hM3Dq-mCherry expression were outlined with 3% opacity. Thus, areas with more hM3Dq-mCherry expression across animals appear more opaque than areas with less hM3Dq-mCherry.

### Quantification and statistical analysis

#### Caspase ablation quantification

Sections were anatomically matched using the Allen Mouse Brain Atlas (mouse.brain-map.org), and then the TN, ARC, lateral hypothalamic area (LHA), and ventromedial nucleus (VMH) were manually outlined in ImageJ. The mean pixel intensity was then measured for each region and normalized to the local background. The mean normalized *Sst* signal in each region was then compared using a 2-way linear mixed-effects model with ablation status (control vs ablated) and region (TN, ARC, LHA, and VMH) as factors and animal as a nested random effect in R (version 4.2.1) using the lme() function (nlme package, version 3.1-157). Following a significant (p<0.05) ablation status x region interaction, we performed multiple t-tests to quantitatively compare *Sst* signal in each region individually. T-tests were performed and p-values calculated using the t_test() function (rstatix package, version 0.7.0), using the Holm method to correct for multiple comparisons.

#### Adipocyte size quantification

Inguinal and white adipose tissue was collected post-mortem and drop-fixed in 4% paraformaldehyde (PFA) for at least 18 hours. Tissue was then washed in PBS before being stored in PBS at 4°C until tissue analysis. For histological processing, tissue was placed in tissue processing cassettes and submerged in 70% ethanol before being embedded in paraffin, sectioned at 4 μM, and stained with hematoxylin & eosin (H&E) by the UCLA Translational Pathology Core Laboratory. Three regions of interest per tissue-type per mouse were imaged by light microscopy at 20x magnification. Adipocyte area was quantified using a custom pipeline in CellProfiler. Inclusion parameters were cell diameters of 100-300 pixel units and a global threshold strategy with minimum cross-entropy.

#### Colocalization analysis

*Sst* and *Esr1* co-expression was determined using CellProfiler (version 4.2.1). First, a contour was drawn around a matched section of the TN using anatomical landmarks (i.e., shape of arcuate nucleus and third ventricle). For each hemisphere, DAPI-stained nuclei were detected and intensity thresholding was used to determine *Sst+* cells. Incorrectly labeled cells were manually erased or added. *Sst+* cells were then filtered based on *Esr1::tdTomato* signal intensity. Counts were made of total *Sst+* cells, as well as *Sst*+/*Esr1*+ and SST+/ *Esr1*- cells. The counts were averaged across the two hemispheres, and percent was calculated as ([*Sst*+/*Esr1*+] / Total SST) x 100.

##### Bioinformatics analysis

*Sst-Cre* female and male mice were bilaterally stereotaxically injected with AAV expressing Cre-dependent tdTomato (see table in surgical procedures). Following at least two weeks for viral expression, animals were sacrificed and TN was microdissected under fluorescent illumination. Dissected TN was dissociated using a papain-based enzymatic process (Worthington Biochemical) and then TN^SST^ neurons were enriched and collected via flow cytometry. Cells were sorted from debris and doublets were excluded by gating on forward-scatter and side-scatter profiles. Live nucleated cells were then selected by DAPI-negative (live) and DRAQ5-positive (nucleated) staining. Finally, tdTomato-positive cells were selected based on relatively high levels of red fluorescence (as in van Veen & Kammel et al., 2020). RNA was isolated from 500-2500 cells by Rneasy Micro kit (Qiagen). Cells were then submitted for bulk RNA sequencing. Single-end reads (∼10 million unique reads per mouse) were assembled to the mouse transcriptome (version mm10) using kallisto (version 0.46.2). Differentially expressed genes and normalized read counts were identified using Deseq2 Galaxy Version 2.11.40.6+galaxy1. Volcano plots were produced by the custom R function “deseq_volcano_plot_gs()” available through the following package: http://github.com/jevanveen/ratplots. Raw reads of the RNA sequencing data were also examined for hypothalamus-peripheral tissue co-correlations across stomach, small intestine, skeletal muscle, visceral fat, and subcutaneous fat as hypothalamic reads using the GTEx database as previously described.^61^^,^^62^ In addition, estrogen-responsive genes used to infer “low” vs “high estrogen signaling were gathered from: https://www.gsea-msigdb.org/gsea/msigdb/cards/HALLMARK_ESTROGEN_RESPONSE_EARLY.html.^113^ To clarify the analysis, estrogen signaling binning per individual and subsequent cross-tissue correlations with mouse DEG orthologues, all processed datasets, scripts used to analyze, and detailed walk-through is available at: https://github.com/Leandromvelez/sex-specific-endocrine-signals.

##### Statistical analyses

All statistics were carried out in R. Sex differences were determined by interaction terms between genotype and sex (caspase ablation experiments) or genotype, treatment, and sex (chemogenetic experiments). In DREADD experiments, data were transformed using the square root function to achieve the assumption of normality necessary for multifactorial ANOVA; raw data are present in Figure 1. In caspase ablation and fat transplantation experiments, animals meeting both the criteria of outlier by Cook’s distance, as well as “miss” (no hit or unilateral hit as defined by more than 5% of targeted cells still present) were excluded. For fat transplantation studies, only sham animals with <10% fat mass at the beginning of the feeding assay were included. All data were checked and transformed, if necessary, to meet normalcy criteria. In all analyses, ANOVA met threshold for interaction prior to running within-group post-hoc t-tests. In linear regressions, ANCOVAs were used. For all figures, ∗p<0.05, ∗∗p<0.01, ∗∗∗p<0.001, and ∗∗∗∗p<0.0001 between ablation groups or r^2^; ^#^p<0.10 and ^####^p<0.0001 within ablation groups.

## Data Availability

• RNA-seq data have been deposited at GEO and are publicly available as of the date of publication. The accession umber is listed in the key resources table. This paper also analyzes existing, publicly available data. Accession numbers for the datasets are listed in the key resources table. All data reported in this paper will be shared by the lead contact upon request. • All original code has been deposited at Github and is publicly available as of the date of publication. Github links are available in the key resources table. • Any additional information required to reanalyze the data reported in this paper is available from the lead contact upon request.

## Supplemental information

Document S1. Figures S1–S3

Table S1. Full details of statistical analyses, related to Figures 1, 2, 3, 4, 6, S1, and S2 ANOVA results and descriptive statistics, including sample sizes for each sex and treatment group, degrees of freedom, F statistics for each factor in the model, and exact p-values, for each experiment.
