# Supplementary material for: Feeding neurons integrate metabolic and reproductive states in mice
Source: iScience. 2023 Sep 14;26(10):107918. doi: 10.1016/j.isci.2023.107918 (PMC10561062; doi:10.1016/j.isci.2023.107918)
Supplement: Document S1. Figures S1–S3 [file mmc1.pdf]

iScience, Volume *26*

Supplemental information

Feeding neurons integrate metabolic and reproductive states in mice

Megan G. Massa, Rachel L. Scott, Alexandra L. Cara, Laura R. Cortes, Paul B. Vander, Norma P. Sandoval, Jae W. Park, Sahara L. Ali, Leandro M. Velez, Huei-Bin Wang, Shomik S. Ati, Bethlehem Tesfaye, Karen Reue, J. Edward van Veen, Marcus M. Seldin, and Stephanie M. Correa

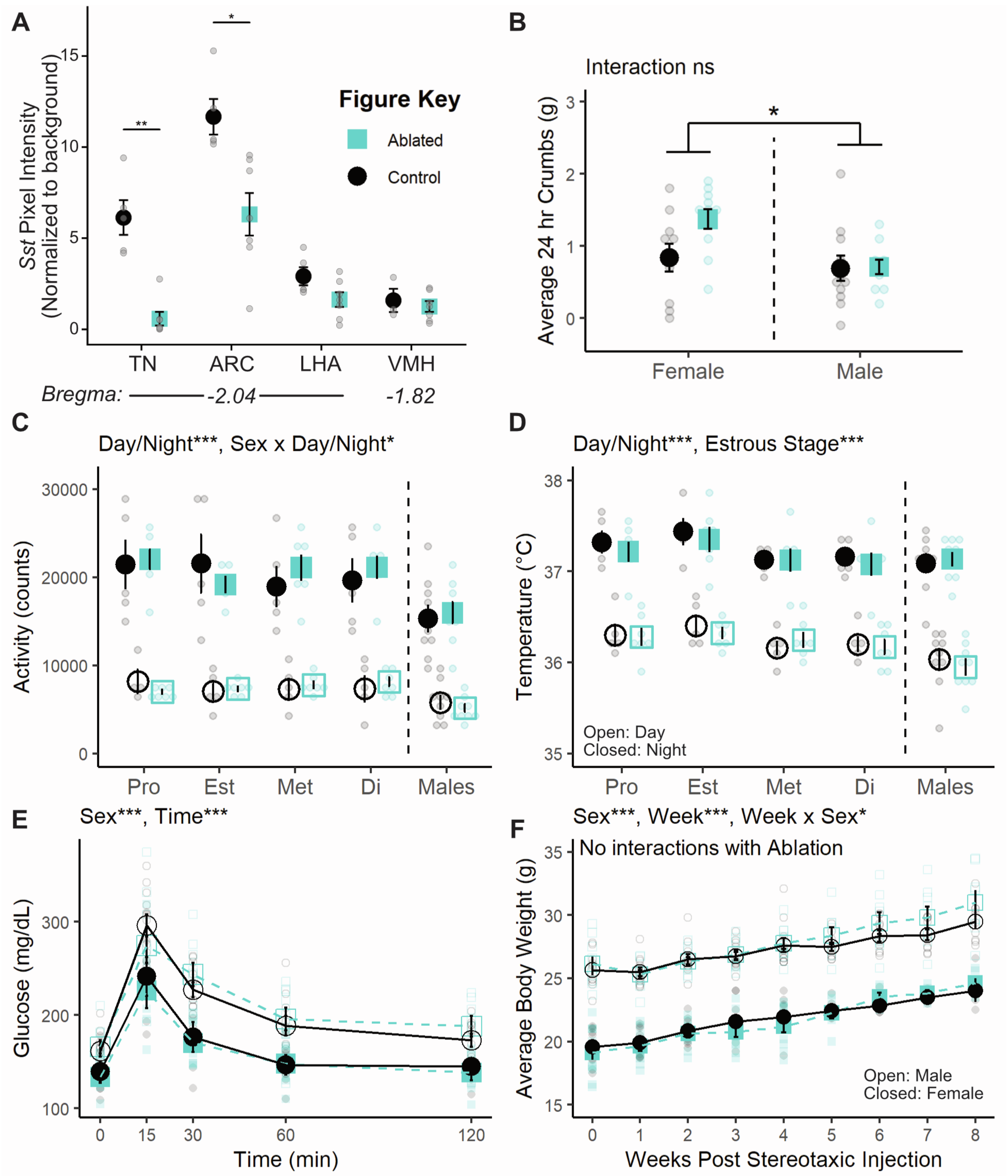

Figure S1. Other metabolic metrics are unaffected by SST neuron ablation, related to Figure 2. (A) Ablation significantly decreased *Sst* staining in the TN and the arcuate (ARC) but did not affect expression in the lateral hypothalamic area (LHA) or ventromedial hypothalamus (VMH). (B) Ablation-induced changes in food intake were not accompanied by changes in gnawing without consumption. Telemetry measures of (C) activity and (D) core body temperature are unaffected by SST neuronal ablation. (E) Fasting glucose tolerance test is also unaffected by SST neuron ablation. (F) Despite changes to food intake, ablation does not affect body weight over time. Mean ± SEM; ANOVA and post-hoc t-tests where applicable; *, p<0.5; ***, p<0.001. M Control n=9; M Ablated n=9; F Control n=5; F Ablated n=7. Pro: Proestrus, Est: Estrus, Met: Metestrus, Di: Diestrus.

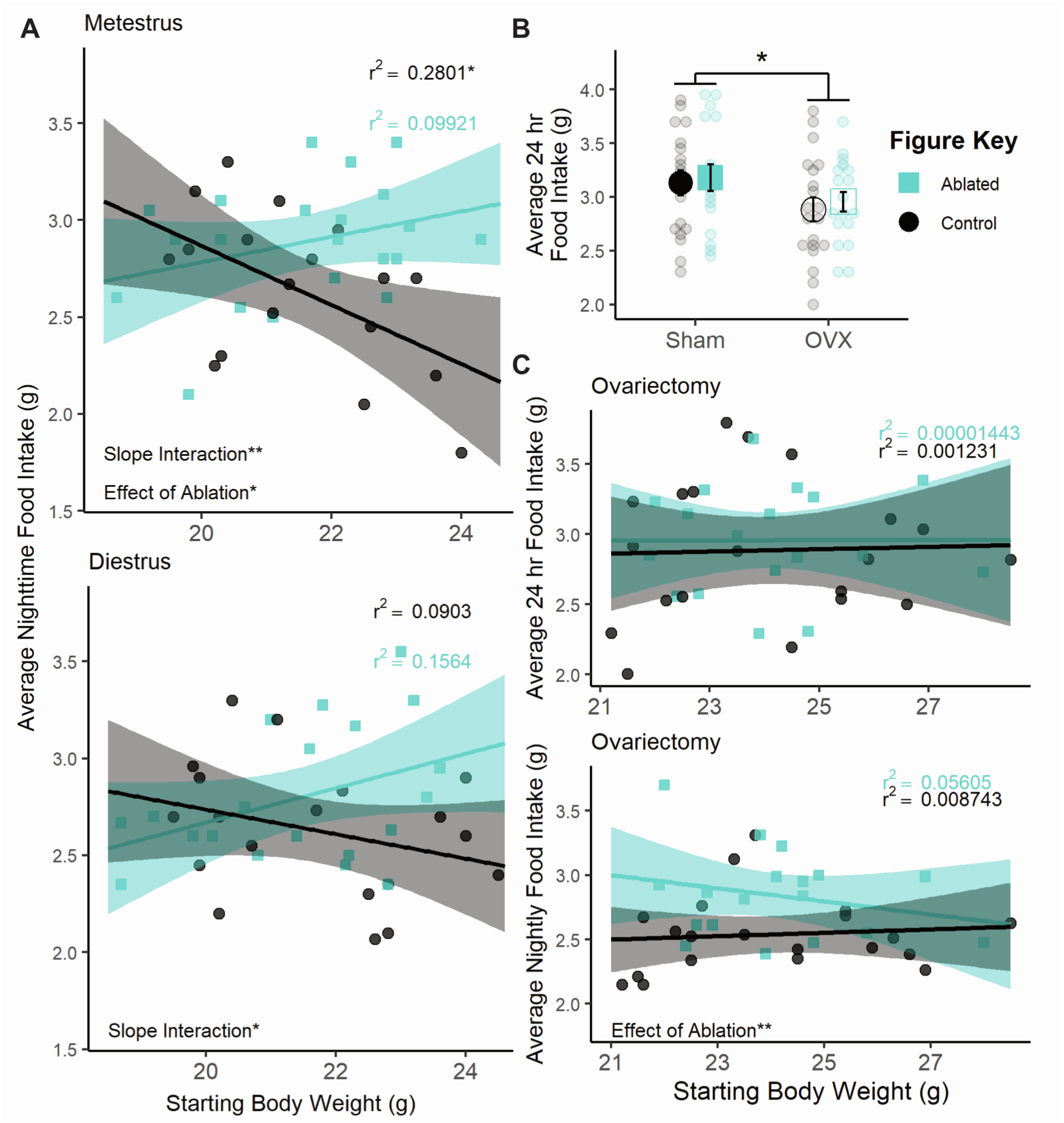

Figure S2. The effect of SST neuron ablation depends on body mass during phases of elevated estradiol but is masked following sham ovariectomy, related to Figure 3. (A) Regression analysis of food intake and body mass across all ovary-intact animals in metestrus (top panel) and diestrus (bottom panel) reveals an interaction between body mass and nightly food intake in females. Significant negative correlations in wildtype animals (black line, round black dots) are seen in the higher estradiol phase of metestrus but not in caspase ablated females (cyan line, square cyan points)**.** Metestrus: Control n=18, Ablated n=21; Diestrus: Control n=19, Ablated n=20. (B) Sham vs. ovariectomy (OVX) in combination with caspase ablation failed to recapitulate ablation-mediated decrease in food intake in sham controls, though OVX mice ate less food than sham controls overall. Mean ± SEM; ANOVA (C) Food intake over 24 hr (top panel) or in the 12 hr dark phase (bottom panel) was not associated with body mass in either ablated or control animals. Sham: Control n=18, Ablated n=16; OVX: Control n=20, Ablated = 18. (A) & (C) Regression lines depict linear regression ± 95% CI; ANCOVA; *p<0.05, **p<0.01

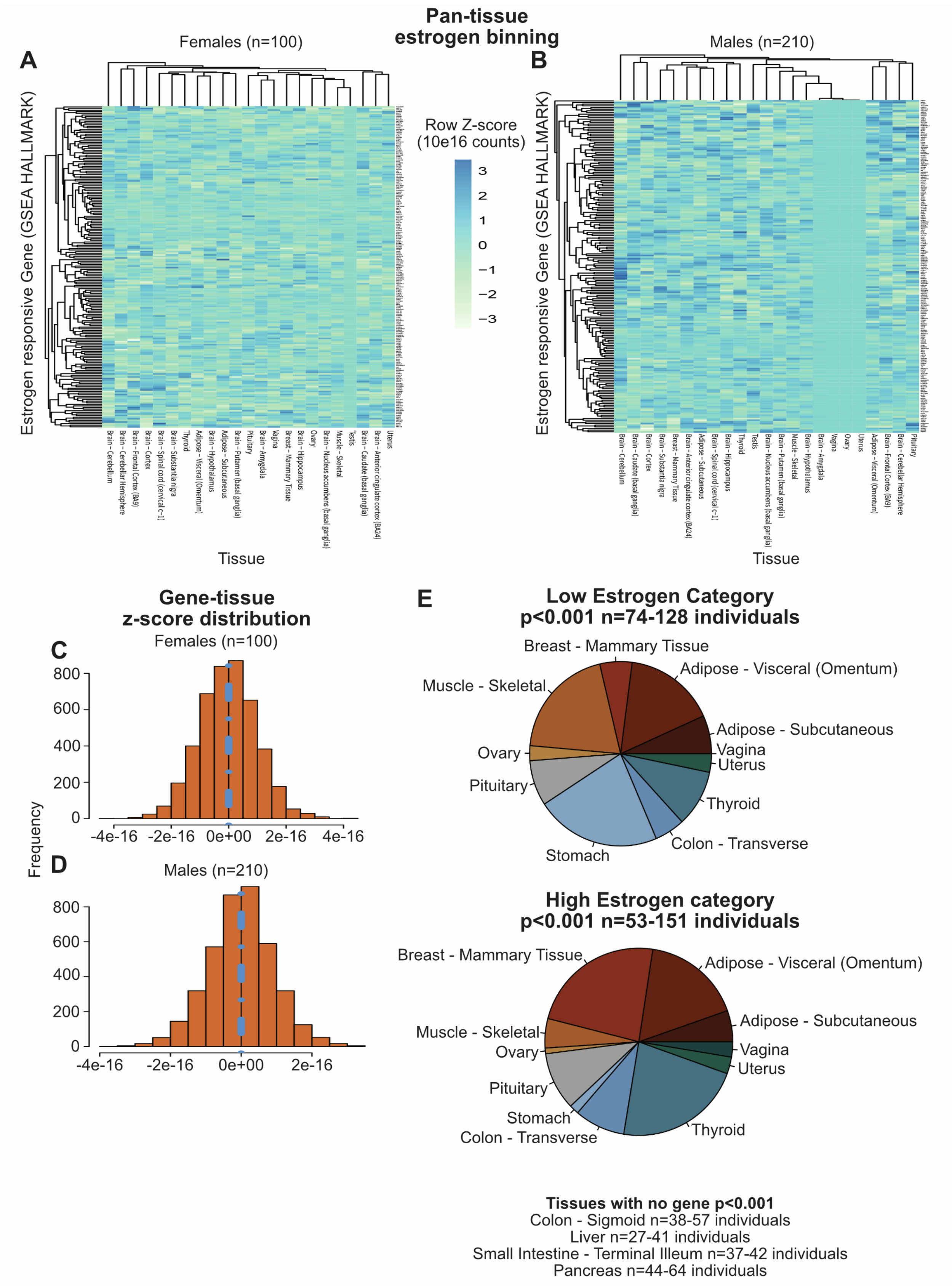
Pan-tissue
estrogen binning
A
Females (n=100)
B
Males (n=210)
Estrogen responsive Gene (GSEA HALLMARK)
Row Z-score
(10e16 counts)
3
2
1
0
−1
−2
−3
Tissue
Gene-tissue
z-score distribution
C
Females (n=100)
D
Males (n=210)
Frequency
800
600
400
200
0
-4e-16
-2e-16
0e+00
2e-16
4e-16
E
Low Estrogen Category
p<0.001 n=74-128 individuals
Breast - Mammary Tissue
Adipose - Visceral (Omentum)
Muscle - Skeletal
Adipose - Subcutaneous
Ovary
Vagina
Uterus
Pituitary
Thyroid
Colon - Transverse
Stomach
High Estrogen category
p<0.001 n=53-151 individuals
Breast - Mammary Tissue
Adipose - Visceral (Omentum)
Adipose - Subcutaneous
Muscle - Skeletal
Ovary
Vagina
Uterus
Pituitary
Stomach
Colon - Transverse
Thyroid
Tissues with no gene p<0.001
Colon - Sigmoid n=38-57 individuals
Liver n=27-41 individuals
Small Intestine - Terminal Illeum n=37-42 individuals
Pancreas n=44-64 individuals

Figure S3. Binning of GTeX individuals into "high" or "low" circulating estradiol levels, related to Figure 5. (A&B) Z-scores of estrogen signaling genes (y-axis, https://www.gsea-msigdb.org/gsea/msigdb/cards/HALLMARK_ESTROGEN_RESPONSE_EARLY.html) across indicated tissues (x-axis) in GTEx female (no Y chromosome, A) and male (Y chromosome present, B) individuals. (C&D) Based on the distributions of these scores across relevant metabolic tissues, individuals were segregated into categories of "low" (<0, left of blue line) or "high" (>0, right of blue line) estrogen signaling and used for cross-tissue genetic correlations. (E) Distribution of DEG co-correlations per tissue in low and high estrogen categories. Tissues with no significant DEG co-correlations tended to have lower sample sizes.
